# Load Dependency of Ventricular Pump Function: Impact on the Non-Invasive Evaluation of the Severity and the Prognostic Relevance of Myocardial Dysfunction

**DOI:** 10.31083/j.rcm2508272

**Published:** 2024-08-01

**Authors:** Michael Dandel

**Affiliations:** ^1^German Centre for Heart and Circulatory Research (DZHK), Partner Site Berlin, 10785 Berlin, Germany

**Keywords:** load dependency of ventricular function, left ventricle, right ventricle, echocardiography, pulmonary hypertension, ventricular remodeling

## Abstract

Ventricular pump function, which is determined by myocyte contractility, preload and afterload, and, additionally, also significantly influenced by heart rhythm, synchrony of intraventricular contraction and ventricular interdependence, explains the difficulties in establishing the contribution of myocardial contractile dysfunction to the development and progression of heart failure. Estimating myocardial contractility is one of the most difficult challenges because the most commonly used clinical measurements of cardiac performance cannot differentiate contractility changes from alterations in ventricular loading conditions. Under both physiological and pathological conditions, there is also a permanent complex interaction between myocardial contractility, ventricular anatomy and hemodynamic loading conditions. All this explains why no single parameter can alone reveal the real picture of ventricular dysfunction. Over time there has been increasing recognition that a load-independent contractility parameter cannot truly exist, because loading itself changes the myofilament force-generating capacity. Because the use of a single parameter is inadequate, it is necessary to perform multiparametric evaluations and also apply integrative approaches using parameter combinations which include details about ventricular loading conditions. This is particularly important for evaluating the highly afterload-sensitive right ventricular function. In this regard, the existence of certain reluctance particularly to the implementation of non-invasively obtainable parameter combinations in the routine clinical praxis should be reconsidered in the future. Among the non-invasive approaches used to evaluate ventricular function in connection with its current loading conditions, assessment of the relationship between ventricular contraction (e.g., myocardial displacement or deformation) and pressure overload, or the relationship between ejection volume (or ejection velocity) and pressure overload, as well as the relationship between ventricular dilation and pressure overload, were found useful for therapeutic decision-making. In the future, it will be unavoidable to take the load dependency of ventricular function much more into consideration. A solid basis for achieving this goal will be obtainable by intensifying the clinical research necessary to provide more evidence for the practical importance of this largely unsolved problem.

## 1. Introduction

Ventricular pump function, which provides the necessary blood supply under a wide range of circumstances to the tissues all over the body, depends in addition to the myocyte contractility, also crucially on the existing loading conditions (i.e., preload and afterload). Furthermore, myocardial pump function is also significantly influenced by heart rhythm, synchrony of intraventricular contraction and ventricular interdependence [1].

Under clinical conditions, evaluation of myocardial contractility is the most difficult challenge because the most commonly used clinical measurements of overall cardiac performance do not differentiate contractility changes from alterations in loading conditions [1]. Under both physiological and pathological conditions, there is a permanent complex interaction between myocardial contractility, ventricular anatomy and hemodynamic loading conditions. During the last 3 decades there has been increasing recognition that a “load-independent index of contractility” cannot truly exist, because loading itself changes myofilament ${\mathrm{Ca}}^{2+}$ sensitivity and force-generating capacity [1, 2]. Therefore, for a reliable evaluation of ventricular dysfunction plus differentiation between predominately myocardial and extra-myocardial causes of that dysfunction, it is necessary to perform multi-parametric evaluations and to use integrative approaches as well as parameter combinations which include details about the ventricular loading conditions.

This article gives an overview of the current knowledge of the relationship between the ventricular pump function and its loading conditions, as well as of the diagnostic tools, with a particular focus on non-invasive approaches, aiming at distinguishing secondary (overloading induced) myocardial morphological and functional alterations from primary myocardial damages. Special attention is also focused on differences between the left and right ventricle (LV and RV, respectively) sensitivity to pressure and volume overloading. Overall, the review aimed to provide an updated theoretical and practical basis for those engaged in this demanding and still current topic due to the new challenges which have arisen especially with the increasing use of temporary or durable mechanical circulatory support devices.

## 2. Definition of Heart Failure: Difficulties and Challenges

Heart failure (HF), a clinical syndrome with different aetiologies and pathophysiology rather than a specific disease, arises from the disability of the heart to pump adequate amount of blood to meet the demands of the body at rest and during physiological effort, without abnormally high cardiac filling pressures [3, 4, 5]. Such inability can result from a complex interplay between intrinsic cardiac abnormalities and extracardiac factors that impair and limit ventricular pump function. Since the early 1990s HF was therefore usually defined as “a pathophysiological state in which an abnormality of cardiac function is responsible for failure of the heart to pump blood at a rate commensurate with metabolic requirements or to do so only from an elevated filling pressure” [3, 4]. Patients who meet this definition of HF are a very heterogeneous group with regard to the underlying pathomechanisms for the pathological reduction of the cardiac output (CO) and/or increase of the filling pressure in both or only in one of the two ventricles (depending on the individual etiopathogenetic particularities of the HF syndrome). More recently, these complex definitions, although accurate in principle, were considered less suitable for the everyday practice because all the requirements can often not be verified in outpatient care, and they also do not apply to all subgroups of patients with HF [6]. Therefore, a recently proposed “universal HF definition and classification”, which aimed to facilitate the evaluation of HF patients, defined HF as a clinical syndrome with symptoms and/or signs caused by a structural and/or functional cardiac abnormality corroborated by elevated natriuretic peptide levels and/or objective evidence of pulmonary or systemic congestion [6]. However, particularly in chronic HF accompanied by signs and symptoms of pulmonary and/or peripheral congestion, further stratification of patients into those with LV and/or RV systolic dysfunction and those with predominantly diastolic dysfunction will often be necessary due to the existence of relevant therapeutic and prognostic differences between these subsets of HF patients [3, 4, 5, 7]. Somewhat surprisingly, in the recently proposed “universal HF definition and classification”, right-sided HF is mentioned only very briefly in a small chapter entitled: “Other syndromes related to heart failure” [6]. As Bozkurt *et al*. [6] underline, they did not specify left- or right-sided HF in their new definition and classification given that in advanced HF biventricular failure is common, and right HF can also be recognized as part of the above definition when patients present with symptoms or signs caused by a cardiac abnormality and have elevated natriuretic peptide levels or objective evidence of cardiogenic systemic or pulmonary congestion. However, the revised classification of HF according to LV ejection fraction (LVEF) which includes HF with reduced LVEF (HFrEF, defined as HF with LVEF ≤40%), HF with mildly reduced LVEF (HFmrEF, defined as HF with LVEF 41–49%), HF with preserved LVEF (HFpEF, defined as HF with LVEF ≥50%), and HF with improved LVEF (HFimpEF, defined as HF with a baseline LVEF ≤40%, a ≥10 point increase from baseline LVEF, and a second measurement of LVEF >40%), as well as the designation of RV failure as only one of “other syndromes related to heart failure” [6, 8], could lead to an underestimation of the impact of RV dysfunction in HF. Last year, in the new American Heart Association/American College of Cardiology/ Heart Failure Society of America guidelines, HF was defined as a complex clinical syndrome with symptoms and signs that result from any structural or functional impairment of ventricular filling or ejection of blood [8]. From a pathophysiological point of view, this definition appears more adequate that the definition proposed before by Bozkurt *et al*. [6].

## 3. Basic Insights into the Pathophysiology of Ventricular Dysfunction

Optimal cardiac function is based on an ordered sequence of mechanical events orchestrated by electrical timing, which involves the interdependent work of both ventricles. Although the LV and RV differ greatly in their size, geometry, architecture, and function, the balance in their outputs must be maintained under equilibrium conditions and also be rapidly restored during or after transitions from one flow rate to another [9, 10]. Ventricular ejection is dependent on myocardial contractility, preload and afterload. Additionally, ventricular pump function is substantially influenced by heart rhythm, cardiac valve function (i.e., valve alterations and dysfunction which affect the loading conditions of the heart), synchrony of intra-ventricular and inter-ventricular contraction, ventricular interdependence and pericardial constraint [10, 11, 12, 13].

Both LV and RV failure occur most often as a consequence of myocardial injury of various causes and/or hemodynamic overloading. In initial stages of ventricular dysfunction, the ongoing increasing stretching of the myocardial fibers by the increased intraventricular end-diastolic pressure (EDP) initiates an adaptive rise in cardiomyocyte contraction force (Frank-Starling law of the heart) and a myocardial hypertrophy. Neuro-hormonal activation (i.e., sympathetic activation) by the low CO, aimed to maintain the blood supply to the vital organs by raising the systemic vascular resistance (SVR) and also the renal retention of salt and water (i.e., renin-angiotensin-aldosterone system activation), also act initially as adaptive responses. Persistent ventricular overloading-induced excessive myocyte stretch and hypertrophy initiate pathological remodeling processes with progressive increase in ventricular wall stiffness and reduction in pump function [14, 15]. Spherical ventricular dilation (i.e., most characteristic remodeling-induced morphologic alteration) increases the systolic wall stress which in turn reduces the efficiency of ventricular myocardial contraction [16]. With the ongoing exacerbation of its geometry alterations the ventricle must develop progressively higher wall tension to preserve the same systolic pressure and, therefore, the dilation itself enhances the mechanical energy expenditure of the failing ventricle. Simultaneously, the progressive increase of the ventricular wall tension, associated with a corresponding reduction of the CO, exacerbates the reduction of the myocardial oxygen supply by impairing the coronary blood flow [15, 16]. Ventricular dilation and remodeling also cause size and geometry alterations of the atrioventricular valve ring which facilitates the development and progression of functional ventricular-atrial regurgitation, inducing ventricular volume overloading with additional reduction of forward stroke volume (_f_SV) [15, 16]. The ventricular volume overloading-induced increase of the end-diastolic volume (EDV) and the reduction of the ventricular relative wall thickness (RWT = wall thickness/cavity diameter ratio) also worsen the functional “afterload mismatch” and can therefore contribute to a further reduction of the CO with an additional increase in ventricular EDP. The reduced ventricular ejection will also increase the end-systolic ventricular volume (ESV), which can further aggravate ventricular dysfunction. The resulting ventricular myocyte overstretching abolishes the Frank-Starling mechanism and also causes a progressive decrease of the myocardial compliance which aggravates the diastolic dysfunction and will have an important contribution to both the severity and the prognosis of HF. Thus, even if a systolic dysfunction was the primary cause of HF, both systolic and diastolic dysfunction will finally contribute together to the symptomatology and severity of HF and cannot be considered as separate and independent entities.

The pathophysiology of diastolic heart failure (DHF) is characterized by a low CO that results typically from a ventricle with thick walls but a small cavity (increased mass/volume ratio) [17, 18]. If the LV is stiff, the slow relaxation in early diastole and the increased resistance to filling in late diastole induce an increase in diastolic pressures associated with reduction of the stroke volume (SV) [18]. The low CO manifests as fatigue, while the high EDP, which is transmitted backwards through the pulmonary veins to the pulmonary capillaries, induces dyspnea under slight physical stress or even at rest [18]. Like in systolic HF, these pathophysiological abnormalities trigger neuro-hormonal activation and increase in the pulmonary vascular resistance (PVR) leading to pressure overload-induced RV dysfunction. In earlier stages, symptoms may be unmasked by exercise because patients with DHF are unable to augment their SV by increasing their ventricular EDV via the Frank-Starling mechanism [18]. These patients often have an exaggerated response of systolic blood pressure to exercise. Mechanisms contributing to abnormal LV diastolic properties include stiff large arteries, hypertension, myocardial ischemia (particularly in patients with coronary microvascular dysfunction without functionally significant epicardial coronary stenoses), diabetes, and intrinsic myocardial changes with or without associated hypertrophy [18, 19].

## 4. Anatomical and Functional Particularities of the Left and Right Ventricle

Certain anatomical and functional particularities of the LV and RV must be considered when assessing their systolic and diastolic function. The most important particularities of the RV are the varying degrees of intensity, spatial direction and timing of regional myocardial contraction, the fact that its different anatomical regions (i.e., inlet, infundibulum and apex) play a different role in blood ejection, as well as its characteristic responses to hemodynamic overloading [20, 21, 22]. In addition, the diversity in myocardial mechanics (including its timing), even among the normal right ventricles, complicates the evaluation of both normal and impaired RV function. Because the thicker sub-endocardial RV layer is composed of preferentially longitudinally arranged myocytes, whereas in the thinner subepicardium (approximately 25% of wall thickness) the myofibers are arranged circumferentially, overall, the RV myocytes are mainly oriented in the longitudinal direction [23]. Due to the different orientations of myocardial fibers in the inflow and outflow part, these two essential parts of the RV are contracting perpendicular to each other: the RV inflow longitudinally and the RV outflow circumferentially [22]. As a result, whereas the normal LV myocardial shortening occurs symmetrically in the transverse and longitudinal planes, the normal RV contraction pattern is mainly characterized by longitudinal shortening [24, 25]. Despite this, chronic pressure and volume overload-induced hypertrophy of circumferential fibers can increase the relative contribution of circumferential myocardial contraction to the global RV systolic function [25, 26]. RV myocardial longitudinal shortening also correlates strongly with RV ejection fraction (RVEF), whereas transverse shortening and RVEF do not correlate [25].

Functioning predominantly as a volume pump, the compliant thin-walled RV tolerates better volume than pressure overload and is more sensitive to afterload changes than the LV [9]. As a consequence of the distinctly high afterload sensitivity of RV pump function, both reductions in ventricular ejection and maladaptive ventricular dilation occur much earlier in the pressure overloaded RV than in the pressure overloaded LV [26, 27].

RV diastolic function also differs considerably from that of the LV. The thin RV walls and the concave interventricular septum (IVS) are relatively distensible conferring the RV a higher compliance which allows greater changes in RV volume, associated with only small changes in the diastolic pressure [25]. Thus, unlike the LV, the RV may dilate significantly in response to acute pressure or volume overload even without a decrease in myocardial contractility [27].

Although both systolic and diastolic myocardial dysfunction are always involved in the pathogenesis of advanced HF due to primary impaired LV function, a particularity of LV dysfunction is that in half of cases, diastolic dysfunction has proved to be the major cause of HF [28, 29]. Before the use of the term HFpEF, this complex clinical syndrome dominated by signs and symptoms of HF despite the absence of relevant LVEF reduction was designated as diastolic HF, and accordingly, HFpEF was originally also considered as a disorder caused solely by abnormalities in LV diastolic function [15, 29]. Meanwhile, there are strong indications that HFpEF should be considered as part of a systemic syndrome involving multiple organ systems, likely triggered by inflammation and with an important contribution of ageing, genetic predisposition, lifestyle factors, and multiple comorbidities [29, 30]. Basic mechanisms affecting the myocardium in HFpEF include myocyte alterations (hypertrophy, diastolic and systolic dysfunction, energetic abnormalities), interstitial fibrosis, inflammation, increased oxidative stress, endothelial dysfunction, as well as reduced density and impaired autoregulation of the microcirculation [7]. The major cardiovascular pathophysiological processes involved in HFpEF incorporate increased systemic vascular resistance, increased conduit arterial stiffness, abnormal ventricular-arterial coupling, reduced LV long-axis systolic function, slowed early diastolic relaxation, reduced LV compliance with increased end-diastolic stiffness, reduced left atrial (LA) reservoir and contractile function, impaired RV function, and chronotropic incompetence [7]. Despite its limitations for predicting cardiac functional reserve and symptoms, the diagnosis of LV failure is still based on LVEF, although in fact, a preserved LVEF has no diagnostic role for HFpEF except to exclude HFrEF [7]. Thus, LVEF enables effective separation of HFrEF and HFpEF patients, but has limited capacity to further stratify HF patients [31]. The LVEF value can estimate global function but does not indicate LV volume or SV [7].

In HF caused initially by LV dysfunction, the rising filling pressures in the LV and LA transmitted to the pulmonary vessels increase the filling pressures in the post-capillary pulmonary circulation and thereby also the PVR. The resulting pulmonary hypertension (PH) associated with increasing RV pressure-overloading will be followed by progressive RV dysfunction.

Left-sided HF-related pulmonary hypertension (PH World Heath Organization [WHO] type 2) is the most common cause of chronic RV failure. Unlike patients with RV failure related to pulmonary arterial hypertension (PAH type 1, precapillary pulmonary hypertension without elevated left-sided heart filling pressures) who benefit particularly from selective pulmonary vasodilatation therapy, those with RV failure related to PH type 2 can often not benefit from such a therapy, which may even aggravate the congestion in the pulmonary circulation if the RV output improvement cannot be immediately balanced by the failing LV.

**Fig. 1.**
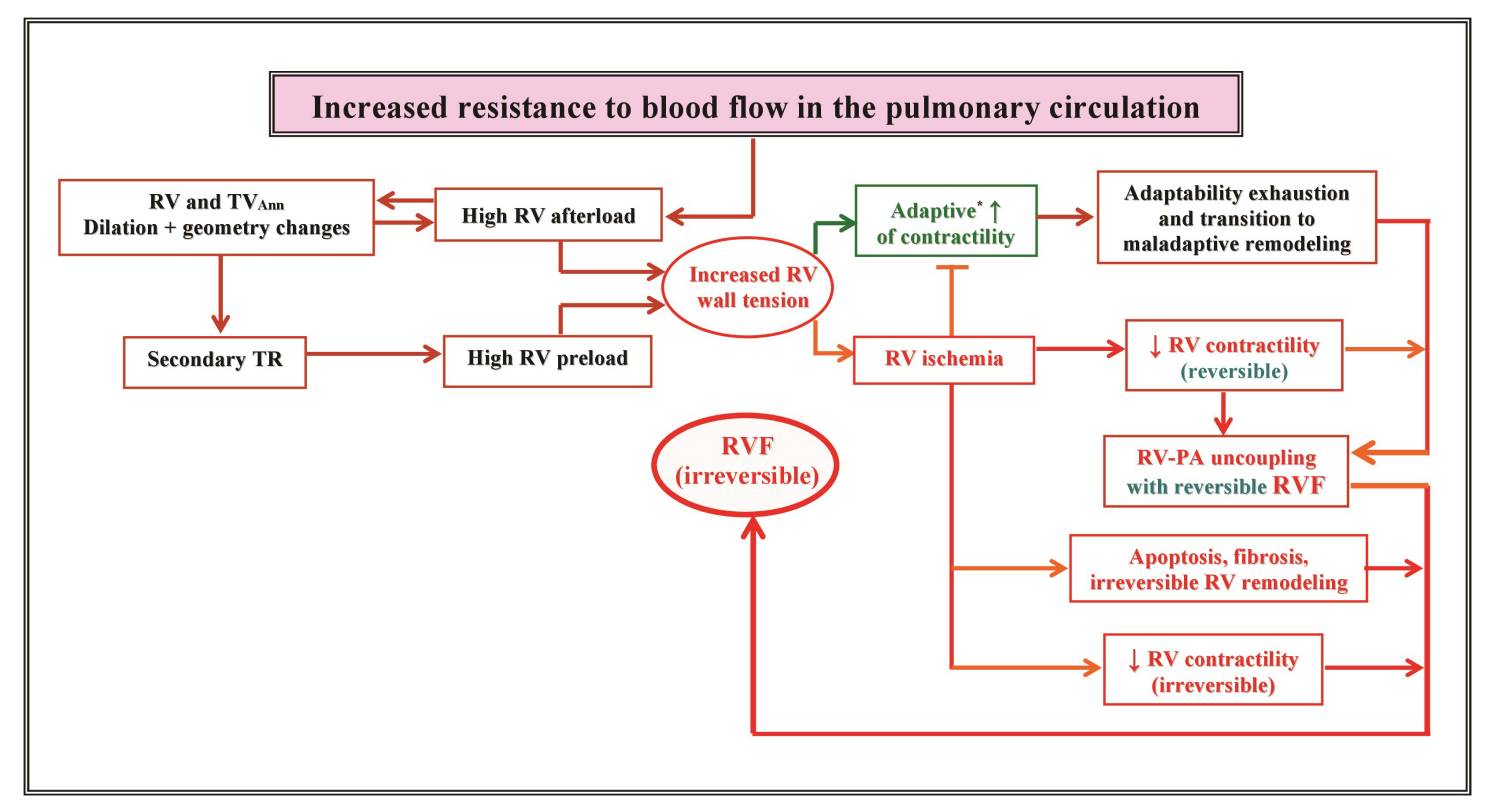
**Overview of pathophysiological mechanisms involved in the development of right ventricular failure secondary to persistent increased resistance to blood flow in the pulmonary ciculation**. RV, right ventricle; ${\mathrm{TV}}_{\text{Ann}}$, tricuspid valve annulus; TR, tricuspid regurgitation; RVF, RV failure; PA, pulmonary artery. The arrows inside the boxes indicate increase (↑) or decrease (↓). Green lettering and green arrows in or outside the box indicates favorable (adaptive) responses. Blue-green lettering in the boxes indicates reversibility of alterations. ^*^ The adaptive response is enabled by the Frank-Starling mechanism and the initially adaptive myocardial hypertrophy.

Given the distinctly high afterload sensitivity of the RV pump function, pressure overload is the predominant pathophysiological mechanism in RV failure and pressure loading resulting from high resistance against blood flow from the pulmonary artery to the LA as well as pulmonary valve stenosis are the major causes for right-sided HF [32]. Other causes of right-sided HF include RV ischemia and infarction, primary cardiomyopathies with mainly RV involvement (e.g., arrhythmogenic right ventricular cardiomyopathy), and cardiac lesions associated with congenital heart diseases. Fig. 1 provides an overview of the pathophysiological mechanisms involved in the development of RV failure in the presence of high resistance to blood flow in the pulmonary circulation.

## 5. Impact of Hemodynamic Overload on Cardiac Structure and Function

It is well established that pressure and volume overload affect ventricular size, geometry and pump function to an extent that depends largely on the intensity and duration of ventricular overloading, as well as on the structural and functional particularities of the LV and RV regarding their resistance and responses (i.e., adaptive or maladaptive) to hemodynamic overloading [9, 26, 33]. Experimental studies revealed that, in pressure overload and volume overload-induced HF, the pathological myocardial remodeling differs not only structurally and functionally but is also associated with distinct proteomic alterations [33].

### 5.1 Left Ventricular Systolic and Diastolic Function during Pressure Overloading 

Pressure overload-induced LV remodeling processes and myocardial dysfunction are a major cause for HF particularly in elderly patients with arterial hypertension (AH) and/or severe aortic stenosis (AS) [34, 35]. For a long time, it has been controversial whether the myocardium preserves normal systolic function in pressure overload LV hypertrophy (LVH) [34]. This resulted in part from the fact that most whole-heart studies incorporated endocardial measurements (e.g., LVEF) that reflect LV chamber function, whereas most experimental studies utilized myocardial or myofibril function [34, 36]. Experimental studies on LV myocardium with pressure overload-induced hypertrophy revealed consistently depressed cardiomyocyte contractility [34, 37]. By contrast, most studies involving the entire LV indicated that the functional state of hypertrophied ventricles evaluated by LVEF measurements remains long time unaltered before the LVEF and other functional indexes become abnormal due to myocardial exhaustion and decompensation [34]. However, the use of LV midwall stress-shortening data strongly indicated that myocardial contractile function in AH-related LVH can be depressed also in the presence of a normal EF [34, 38, 39, 40]. Comparing patients with massive AH-related pressure overload LVH but undoubtedly normal LVEF with healthy persons (LVEF: 69 ± 13% and 63 ± 11%, respectively, *p* < 0.01), Aurigemma *et al*. [34] found in the AH patient group a depressed LV myocardial shortening at the midwall and along the long axis, which indicated the presence of a relevant myocardial contractile dysfunction. They concluded that in patients with hypertensive LVH, the indexes of LV chamber function (ejection fraction and circumferential shortening at the endocardium) may be normal or even increased in the presence of depressed midwall and long-axis shortening. They also found that the difference between endocardial and midwall shortening was directly related to the magnitude of LVH, reflected by the RWT whose increase reduces the LV systolic wall tension [34]. Their observations suggest that the increased endocardial circumferential shortening in the AH-related pressure overload LVH patient group in comparison to healthy persons (42 ± 10% vs. 37 ± 5%, *p* < 0.01) could be an adaptive myocardial response to the high afterload [34]. These considerations underscored the impact of ventricular geometry on endocardial shortening, and they suggest that an analysis of LV myocardial shortening at the midwall and along the long axis is particularly important when LV mass and geometry are changing, especially for the evaluation of LVH regression during the treatment of AH patients, as well as for the postoperative patient monitoring after aortic valve (AV) replacement for AS [34, 40]. The speckle-tracking-derived myocardial strain imaging by echocardiography (also known as speckle-tracking echocardiography [STE]), which was introduced in the clinical praxis after 2005, has meanwhile unambiguously confirmed the higher sensitivity of myocardial deformation analysis in comparison to the LVEF measurements for early detection and more reliable grading of LV systolic dysfunction [41, 42, 43, 44].

The adaptive responses of the LV myocardium to pressure overload appear to be both highly relevant and strikingly reversible even at advanced ages. An evaluation of 80 patients (mean age 80 ± 11 years) with severe AS and high surgical risk before transcatheter AV implantation (TAVI) revealed at 8 ± 3 months after TAVI an improvement of LVEF only in those with LVEF <50% before TAVI (EF increase from 34.7 ± 10% to 49 ± 13%, *p* < 0.001), although in this group the prevalence of coexistent coronary artery disease (CAD) was almost twice as high than in the group with pre-implantation LVEF ≥50% (76.5% vs. 39.1%, *p* < 0.001) [45]. Although in that study there was a significant LVEF and SV increase only in the patient group with LVEF <50% before TAVI, the systolic pulmonary arterial pressure (PAPS) decreased significantly and the tricuspid annular plane peak systolic excursion (TAPSE) increased significantly in both groups [45]. This indicates a reduction of left-sided heart filling pressures after TAVI and thus also the improvement of LV diastolic function which reduces both the congestion in the pulmonary circulation and the pressure overloading of the RV. Also, in the study by Naeim *et al*. [45], STE data revealed in both patient groups important beneficial post-TAVI changes in myocardial mechanics, which were partially different, although before TAVI the mean values of the stenotic AV areas were nearly identical in the two patient groups. Thus, whereas the global LV longitudinal strain and strain rate (GLS and GLSR) improved significantly in both groups, the global circumferential strain and strain rate (GCS and GCSR), which were at baseline significantly lower in the patients with LVEF <50%, increased significantly in this group after TAVI, while in the group with LVEF ≥50%, their values showed after TAVI an insignificant tendency toward lower values [45]. Strikingly, the apical circumferential strain (ACS), which increased in the group with reduced LVEF from depressed values (18.7 ± 11%) towards normal values (22 ± 12%; *p* < 0.03), decreased in the group with preserved LVEF from supra-physiological values (36 ± 11.5%) towards normal values (32 ± 9%; *p* < 0.024). Similarly, the net LV twist angle, which was low in patients with LVEF <50%, increased significantly after TAVI towards normal values, whereas in those with preserved LVEF, where this parameter was supra-physiological before TAVI, decreased significantly thereafter [45]. These observations suggest that like in AH-related pressure overload LVH, high (supra-physiological) endocardial circumferential shortening could be an adaptive myocardial response to the high afterload. At least equally important is also the evidence provided by Naeim *et al*. [45], that the alterations in myocardial mechanics detectable in patients with severe AS are at least partially reversible after elimination of the stenosis even in octogenarian persons, and even in those with coexistent CAD and evidence for reduced contractile function. This observation not only confirms the assumption that many of the myocardial responses to pressure overload are potentially reversible adaptive responses, but also underscores the benefits of AV replacement even in elderly persons, for which a coexistent CAD should not be considered by no means as an absolute contraindication for TAVI.

As time passes, pressure overload-induced LV hypertrophy will be accompanied by progressive diastolic dysfunction due to delayed relaxation and interstitial fibrosis-related increased LV chamber stiffness [46]. In a study of AS patients who have not undergone valve surgery, the mitral E/e’ ratio (i.e., ratio of early diastolic mitral inflow velocity to early diastolic mitral annulus velocity) was the most predictive parameter of clinical events among clinical and imaging measurements [47]. In other studies, the E/e’ ratio was an important independent predictor of early, midterm, and late mortality after AV replacement [46, 48].

### 5.2 Left Ventricular Systolic and Diastolic Function during Volume Overloading

LV volume overload occurs more often in response to mitral regurgitation (MR) or aortic regurgitation (AR) where a relevant part of the ejected blood is not delivered to the systemic circulation, but instead is either delivered back to the LA or returned to the LV, respectively. The chronic volume overload results in LV chamber enlargement with eccentric myocardial hypertrophy which allows the ventricle to counteract, at least in part, the negative impact of the regurgitation on the _f_SV [21].

LV volume overload is the major pathognomonic feature of chronic AR. The degree of volume overload is determined by the magnitude of the AR, which is related to the size of the regurgitant orifice, the aorta-ventricular pressure gradient, and the diastolic time [21]. However, the AR-related LV volume overload induced by the simultaneous increase of both the SV and the regurgitant volume (RegV) associated with concomitant progression the LV eccentric myocardial remodeling lead inevitably also to an increase in LV myocardial wall tension, and thus also to an additional pressure overloading. The progressive increase of the LV end-diastolic volume (LVEDV) can also induce dilation and geometry change of the mitral valve ring associated with MR of different degrees which aggravates LV dysfunction by aggravation of the volume overload [21].

### 5.3 Right Ventricular Systolic and Diastolic Function during Pressure Overloading

The adaptation of the RV to pressure overload is based on its intrinsic myocardial contractility, the duration, progression rate (e.g., chronic steadily increase or acutely occurring massive increase in the resistance to blood flow in the pulmonary circulation) and severity of the pressure overloading, as well as the adaptability of the RV myocardium to sustain abnormally high wall stress [49, 50]. The initial adaptive responses to persistent pressure overload, which are mainly achieved by an increase in myocardial mass (i.e., adaptive hypertrophy) and contractility, are enabled by an upregulation of subcellular organelles (e.g., sarcolemma, sarcoplasmic reticulum, myofibrils, and mitochondria), which aim to minimize the wall stress for the RV exposed to an abnormally high workload (homeometric adaptation) [51, 52]. In this early adaptive state, RV–pulmonary artery (PA) coupling, the CO and the RVEF, as well as the exercise capacity are maintained [51]. However, myocardial hypertrophy leads to increased RV diastolic pressure, which indicates that the increased RV contractile function occurs at the cost of an alteration of its diastolic function [51]. The aggravation of pressure overloading finally exhausts the homeometric adaptation and induces a transition of the RV alterations to a heterometric adaptation where the RV dilates, and uncoupling arises because its contractility fails to match the excessively high afterload [51].

There are different stages of adaptive and maladaptive RV remodeling processes. Throughout maladaptive RV remodeling, there are further stages of reversible and irreversible RV failure (RVF). Thus, adaptive and maladaptive phenotypes are not completely different responses, but rather parts of a sequence of ventricular responses to pressure overloading [53]. Currently, the mechanisms behind the transition toward RVF are still incompletely understood.

Several mechanisms and myocardial structural changes have been associated with either adaptive or maladaptive RV phenotypes (e.g., sympathetic hyperactivity, metabolic shift from oxidative metabolism toward glycolysis, capillary rarefaction, and fibrosis). Experimental data suggest a larger contribution of interstitial fibrosis to total stiffness in end-stage RV failure, whereas cardiomyocyte stiffening may play a larger role in earlier stages of pressure overload-induced RV structural and functional alterations [54]. Prolonged activation of adaptive mechanisms, particularly in association with a progressive increase of pressure overloading, finally leads to severe RV systolic dysfunction and diastolic stiffness, followed by irreversible RVF. Given that RV adaptation to pressure overload is quite variable among patients, the progression to right-sided heart failure remains difficult to predict [53].

After full exhaustion of its adaptive capacities to overcome the excessively high resistance in the pulmonary circulation, the RV responses to the continuously high afterload exhibit more often a transition to pathological myocardial hypertrophy associated with a down-regulation of subcellular adaptive activities as well as ischemia due to reduced capillary density [52, 53, 54]. Oxidative stress, ${\mathrm{Ca}}^{2+}$-handling abnormalities, mitochondrial dysfunction, inflammation, and cardiomyocyte apoptosis appeared particularly involved in the alteration of contractile function in patients with pathological RV hypertrophy [49, 50, 55].

RVF caused by ventricular pressure overloading is ultimately the consequence of both systolic and diastolic RV dysfunction. Nevertheless, whereas RV systolic dysfunction was steadily found to be an independent predictor of mortality in PH, for RV diastolic dysfunction such correlation was not found in all studies [56, 57]. Given that RV diastole consists of several phases, it cannot be characterized by one single parameter and, therefore, the evaluation of RV diastolic function is much more demanding [9].

### 5.4 Right Ventricular Systolic and Diastolic Function during Volume Overloading

Because of its anatomical and physiological particularities, the RV tolerates volume overload better, and for much longer periods of time, than pressure overload [58]. Whereas in children the most common causes of RV volume overload are congenital heart diseases, in adults, tricuspid and/or pulmonary regurgitation in the presence of various cardiac pathologies are the major triggers of RV chronic volume overload [59]. In the early stages of volume overloading, the RV increases its contractile function through the Frank-Starling law, which can effectively compensate for the altered hemodynamic conditions. This adaptive response was also confirmed by the evidence of an increase in RV longitudinal shortening in the presence of relevant volume overload [59]. Chronic volume overload may ultimately lead to RV systolic dysfunction and increased morbidity and mortality, particularly in the presence of superimposed pressure overload and/or marked RV enlargement, which argues for corrective interventions before significant RV dilatation ensues [58, 60]. RV volume overload leads also to simultaneous LV dysfunction, without intrinsic alteration in myocardial contractility, primarily due to LV underfilling secondary to septal displacement and changes in LV geometry rather than due to a decreased _f_SV of the RV [60].

## 6. Impact of Loading Conditions on the Evaluation of Ventricular Function

Ideally, an index of contractility should be sensitive to changes in myocardial contractile state but indifferent to loading conditions [61]. Already more than 3 decades ago it was proven that the EF, which was long considered the most useful single hemodynamic parameter for the assessment of ventricular dysfunction, is highly load dependent and, therefore, its usefulness as a measure of ventricular function is limited [61, 62]. Because the EF reflects the ventricular contractile function in relation to loading conditions, it cannot be considered as an index of contractility [9]. Accordingly, patients with identical EF values can have very different levels of myocardial contractility and ventricular dysfunction [9]. Even with unchanged contractility an increase of the afterload can relevantly reduce the EF, whereas a reduction of the afterload increases the EF [63, 64, 65, 66, 67]. LVEF can be normal in patients with LV hypertrophy associated with small cavity size, even in the presence of LV systolic dysfunction [65]. Regarding the highly load-sensitive RV, because there is no single morphological or functional cardiac parameter which can alone reveal the overall appearance of RV morphological and functional alterations, it is necessary to conduct multiparametric assessments and to use integrative approaches utilizing parameter combinations which also include data referring to the RV actual loading conditions [9, 68, 69, 70].

### 6.1 Left Ventricular Evaluation in Relation to Loading Conditions

There are several clinical presentations of LV dysfunction where the commonly used volumetric EF calculation: LVEF (%) = [(EDV – ESV)/EDV)] × 100, based on EDV and ESV measurements enabled by different non-invasive methods (e.g., echocardiography [Echo], magnetic resonance imaging [MRI], computed tomography [CT], or invasively by LV contrast ventriculography during catheterization, does not reflect the fraction of chamber volume ejected into the systemic circulation [21, 63, 71]. In this regard, both MR and AR are of highly important relevance.

In the presence of MR, the difference between the EDV and ESV does not reflect anymore the _f_SV because it becomes the sum of _f_SV + RegV [21, 63, 72]. This leads to an increase of the volumetric LVEF corresponding to the increased blood volume delivered back to the LA which, in turn, erroneously leads to overestimation of LV contractile function [21, 65]. Such overestimation can result in an unfavourable delay of a necessary mitral valve (MV) replacement or repair. Overestimation of LV pump function in the presence of relevant MR can be avoided by using the formula: LVEF (%) = [_f_SV/EDV] × 100. Direct measurements of the _f_SV are possible with the pulsed-wave Doppler [63, 64, 73]. Such measurement of the _f_SV also allows calculations of the RegV and the regurgitant fraction (RegF) [66, 73, 74]. The mitral RegV which is often more difficult and less reliably and directly measurable can be calculated from the directly measured LV EDV, ESV and _f_SV by the formula: RegV = EDV – (ESV + _f_SV) [72, 73, 74]. The RegF can be obtained by dividing the RegV by the total volume ejected by the LV during systole (i.e., _f_SV + RegV). Given that in the presence of MR the difference between the EDV and ESV becomes the sum of _f_SV + RegV, the RegF can also be calculated by the formula: RegF (%) = [RegV/(EDV – ESV)] × 100 [74, 75].

Although the LVEF reflects the LV contractile function also in the presence of AR, it is important to take into consideration that an increase of the aortic RegV in the detriment of the delivered blood volume into the systemic circulation will not change the LVEF as long as the amount of blood ejected into the aorta (i.e., _f_SV) remains unchanged. In the presence of AR, the true effective blood volume which ultimately leaves the LV (i.e., the difference between the _f_SV and RegV) can therefore differ even between patients with identical LVEF, but different degrees of AR (i.e., different RegF). Thus, LVEF lacks sensitivity to detect subclinical LV dysfunction in patients with AV disease [76]. This also explains why in asymptomatic patients, the LV cavity size measurements were found more useful than the LVEF value for the prediction of postoperative outcomes and for decision-making regarding the necessity of AV replacement [75, 77]. The development of a secondary MR as a consequence of the AR-related volume overloading-induced LV dilation can furthermore misleadingly increase the volumetrically calculated LVEF [21].

In patients with AS, LVEF reduction does not clearly suggest impaired myocardial contractility, if the hypertrophied left ventricle can develop supernormal systolic pressures without relevant changes of its cavity size and geometry [77, 78]. This can explain the frequently detected EF improvement after AV replacement [78]. Given the increasing prevalence of AS with advancing age, as well as its more frequent association with atherosclerosis and AH in these patients, the impact of reduced systemic arterial compliance (SAC) on the LV afterload in patients with “degenerative” AS was more intensively investigated during the last two decades [79, 80, 81, 82]. Meanwhile there is conclusive evidence that in AS patients older than 60 years, particularly in those with low flow AS associated with reduced SAC, the LV faces a double load (i.e., valvular and arterial) and, therefore, AS cannot be viewed in all patients as an isolated disease of the AV [79, 80, 81]. In patients with AS, reduced SAC has a major influence on the occurrence of LV systolic and diastolic dysfunction [81]. Relevant AS and reduced SAC showed additive effects in increasing afterload and deteriorating LV function [80, 82]. The total SAC can be indirectly calculated with the equation: SAC = SVi/PP, where SVi is the SV index, and PP the brachial pulse pressure (i.e., the difference between systolic and diastolic arterial blood pressure). Whereas SVi/PP >0.6 mL/$m^{2}$/mmHg indicates normal SAC, values ≤0.6 mL/$m^{2}$/mmHg indicate reduced SAC [80]. For estimation of the global LV afterload in AS patients it appeared useful to calculate the “valvulo-arterial impedance” ($Z_{\text{va}}$) according to the formula: $Z_{\text{va}}$ = (SAP + ${\mathrm{MG}}_{\text{net}}$)/SVi, where SAP is the systolic arterial pressure, ${\mathrm{MG}}_{\text{net}}$ is the mean net gradient across the narrowed AV (i.e., mean gradient taking into account pressure recovery) and SVi is the SV index [80, 81]. $Z_{\text{va}}$ represents the valvular and arterial factors that oppose LV ejection by absorbing the mechanical energy developed by the LV. A $Z_{\text{va}}$ ≥5.0 mmHg/mL/$m^{2}$ indicates increased afterload that exceeds the limit of LV compensatory mechanisms and, therefore, leads to afterload mismatch with subsequent LV systolic dysfunction [80]. From a therapeutic point of view, it is of crucial importance to determine the respective contributions of the AV and the SAC to the afterload excess because even if the high SAC cannot be normalized by medical treatment, the implantation of AV prosthesis can be beneficial for the patient if this would result in a significant reduction in the LV afterload. In this regard it appeared useful to confront the value for $Z_{\text{va}}$ to the values of the energy loss index (ELI) and SVi/PP [80]. The ELI, a parameter calculable by the formula: ELI = [(EOA × $A_{\text{A}}$)/($A_{\text{A}}$ – EOA)]/BSA, where EOA is the effective orifice area of the AV, $A_{\text{A}}$ is the aortic cross-sectional area calculated from the diameter of the aorta measured at the sino-tubular junction, and BSA is the body surface area, appeared useful for a better classification of patients with AS, based on both the severity of AV narrowing (severe AS defined as ELI ≤0.55 ${\mathrm{cm}}^{2}$/$m^{2}$) and the degree of SAC alteration [80]. Comparing 2 groups of elderly patients with severe AS (ELI >0.55 ${\mathrm{cm}}^{2}$/$m^{2}$) and similar AV area indexes (i.e., 0.39 ± 0.06 and 0.39 ± 0.11 ${\mathrm{cm}}^{2}$/$m^{2}$) but with significantly different SAC (one group with SVi/PP values >0.6 mL/$m^{2}$/mmHg, indicating normal arterial compliance, the other with SVi/PP values ≤0.6 mL/$m^{2}$/mmHg, indicating reduced arterial compliance), in the group with reduced arterial compliance, the $Z_{\text{va}}$ (which reflects the global afterload) was significantly higher, whereas the peak and mean pressure gradients across the stenotic AV, as well as the SV and cardiac index, were significantly lower [80]. In multivariate analysis excluding $Z_{\text{va}}$ identified $Z_{\text{va}}$, both the ELI and the SAC appeared to be independent predictors of LV dysfunction, but when $Z_{\text{va}}$ was entered into the analysis, it become the only hemodynamic variable associated with LV diastolic and systolic dysfunction [80]. Also in that study, although the global afterload was significantly higher in the group with pathologically reduced arterial compliance, the global afterload in that group was only 28.6% higher than in the group with normal arterial compliance (i.e., 5.4 ± 1.1 vs. 4.2 ± 0.7 mmHg/mL/$m^{2}$, *p* < 0.001), which indicates that a LV afterload reduction by AV prosthesis implantation can be beneficial also in the presence of reduced SAC [80].

Because LVEF reduction is associated with poor prognosis in patients with relevant AS, a progressive EF reduction is considered as a strong indicator for necessary intervention [83]. However, in patients without additional cardiac co-morbidities, several adaptive mechanisms which act to preserve the EF can delay the fall in EF during the transition period toward LV failure. This was confirmed by the use of STE, which appeared more reliable than the conventional echocardiography for early identification of patients with AS at great risk for LV failure [84, 85].

A more recent attempt to improve the diagnostic and prognostic value of the LVEF revealed that the EF up to the time of maximal ventricular fiber shortening named as: “First-Phase Ejection Fraction” (${\mathrm{EF}}_{1}$), can predict cardiac worsening in patients with ≥moderate AS better than other parameters including the global longitudinal strain, the aortic valve area index, mean pressure gradient, SV index and trans-aortic flow rate [83]. ${\mathrm{EF}}_{1}$ appeared also helpful for the timing of a valve replacement in asymptomatic patients with severe AV narrowing [83].

Due to the load dependency of all the measurements which are routinely used in daily practice for evaluation of LV systolic function it is necessary to consider this important problem in the interpretation of measured values [21, 63]. However, the evaluation of systolic function in relation to its loading conditions requires integrative approaches using several LV anatomical and functional parameters in combination with hemodynamic parameters which usually necessitate both invasively and noninvasively obtained measurements. However, such demanding integrative approaches which include also invasively obtained hemodynamic parameters are hardly applicable in the outpatients. The introduction of new completely non-invasive methods for quantifying the LV myocardial work (MW), which take into account the loading conditions during myocardial deformation and involve combining non-invasively obtained LV pressure curves with STE-derived strain measurements, has resulted in rising interest for noninvasive integrated approaches [86, 87, 88, 89, 90, 91]. The pressure curve can be obtained by associating peripheral systolic blood pressure (SBP) with Echo-derived cardiac event times including isovolumic contraction (IVC), systolic ejection, and isovolumic relaxation (IVR) [88, 90]. With software support, by integrating the STE-derived myocardial longitudinal strain measurements with the pressure curves, it is possible to generate a non-invasive LV pressure-strain loop, which enables the quantification of MW [88, 90].

MW consists of 4 distinct components: the global work index (GWI), the global constructive work (GCW), the global wasted work (GWW), and the global work efficiency (GWE) [87, 88, 89, 91]. Each of these components provides different information about LV mechanics. The GWI quantifies the indexed total work performed by the LV throughout the entire mechanical systole including both IVC and IVR (corresponds to the myocardial energy translated into mechanical energy between MV closure and opening) [87]. The GCW is the LV work that contributes to LV ejection and is performed by contraction with and without myocyte shortening (i.e., positive work during the IVC and ejection, respectively), and by active relaxation (negative work implying energy-dependence) with and without myocyte lengthening during the IVR and the early diastolic LV filling, respectively [87]. Thus, the GCW quantifies the energy consumed by the myocardium that contributes effectively to the generation of the CO by facilitating LV ejection. On the other hand, GWW represents the negative work that does not contribute to LV ejection [87]. It includes the negative segmental work during IVC and ejection where the myocardium undergoes lengthening as well as positive segmental work during IVR when the myocardium undergoes shortening. It quantifies the energy consumed by the myocardium that is wasted and does not contribute to CO. Finally, the GWE is the ratio between constructive work and total (constructive and wasted) work, which reflects the net percentage of performed MW which is actually translated into CO and can be calculate with the formula: GWE = [GCW/(GCW + GWW)] × 100 [87, 88]. In one study, patients with arterial hypertension (SBP >160 mmHg) revealed significantly higher GWI values in comparison to healthy persons, despite rather unexpectedly normal GLS values [87]. This result confirms that usual STE-parameters as GLS are alone not able to reflect the increased cardiac energy demand to counteract increased afterload.

In AS patients, the estimation of LV pressure from brachial artery blood pressure measurements is limited due to the presence of AV obstruction, which creates a higher peak intraventricular pressure compared with the peripheral SBP. To address this limitation, Fortuni *et al*. [90] introduced a method in which they combined the mean gradient over the AV measured by echocardiography with arterial SBP, creating a new parameter for the noninvasive estimation of the LV pressure in patients with AS. GWI and GLS tend to vary over time in AS patients. Initially, as long as the AS is compensated and LV contractile function is preserved, both GWI and GCW increase, whereas absolute GLS can appear incorrectly low due to its afterload dependency [91]. Later, in decompensated stages, when LV contractile function becomes impaired, both GWI and GCW decrease, symptoms of heart failure occur irrespective of the underlying GLS [91]. Even in asymptomatic AS patients, lower GWI values appeared to be a marker of decompensation and was also found associated with increased mortality [91, 92].

In AR, which is characterized by an increase in both pre- and afterload, initially, the RegV reduces the final SV which leaves the LV and increases the LV enddiastolic volume. The elevated preload triggers LV adaptation according to the Frank-Starling law which leads to an increase in SV that tends to compensate for the volume overload. The elevated preload triggers LV adaptation according to the Frank-Starling law which leads to an increase in SV that tends to compensate for the volume overload. In the further course of the AR, sustained high LV wall tension progressively leads to increased afterload. A recent study which included patients with moderate or severe chronic AR and preserved LVEF has revealed that before surgical intervention, both GWI and GCW were elevated in relation to the severity of AR [93]. A recent study which included patients with moderate or severe chronic AR preserved LVEF has revealed that before surgical intervention, both GWI and GCW were elevated in relation to the severity of AR [93]. After the elimination of the AR, the GWI, GCW, and GWE decreased significantly, while GWW remained unchanged [93]. However, in 28% of the patients, the GWI remained abnormally high, suggesting reduced LV reverse remodeling in the presence of irreversible myocardial damage [93].

Patients with severe secondary MR revealed significantly altered GWI and GCW values [94]. However, those alterations appeared to be associated with better GWE and better preserved (i.e., less impaired) GWW values, which suggest the existence of potential benefits on myocardial energetics caused by the additional low impedance alternative for a partial emptying of the LV into the LA [94]. This could explain the finding that not only the altered GWI and GCW, but also better GWE and better preserved (i.e., less impaired) GWW values appeared independently associated with worse long-term survival in those patients [94]. The latter may also suggest that in very advanced stages of LV remodeling and dysfunction, associated with severe secondary MR, the elimination of MR (by MV repair or replacement) could also offset the potential benefits on myocardial energetics provided before that therapy by the low impedance LA leak through the incompetent MV, could trigger the development of post-interventional acute life-threatening LV decompensation. It is well known that the postoperative necessity of a ventricular assist devce (VAD) implantation or heart transplantation in patients with advanced LV failure who underwent MV surgery for severe secondary MR is not an absolute rarity [95]. Table 1 (Ref. [21, 63, 64, 65, 66, 72, 73, 74, 75, 79, 80, 81, 82, 83, 86, 87, 88, 89, 90, 91]) provides an overview of major Echo-derived combined parameters and indices for evaluation of LV myocardial responses to pressure and/or volume overloading.

**Table 1.** **Summary of the most recommended echocardiography-derived parameter combinations and indices for evaluation of myocardial responses to left ventricular pressure and/or volume overloading**.

Parameters	Calculation	Particularities and clinical usefulness
MR-corrected LVEF [21, 63, 64, 65, 66, 72, 73, 74, 75]	LVEF (%) = [_f_SV/EDV] × 100	- Avoids overvaluation of LV pump function in the presence of mitral regurgitation (MR) because the difference between the measured EDV and ESV is not anymore only the blood volume rejected into the aorta, but in fact becomes the sum of _f_SV and RegV.
	_f_SV = forward stroke volume	- _f_SV measurements are possible with the Doppler. The RegV and RegF can be calculated by the formulas: RegV = EDV – (ESV + _f_SV) and RegF (%) = [ RegV/(EDV – ESV)] × 100.
	EDV = end-diastolic volume	
	RegV = regurgitant volume	
	RegF = regurgitant fraction	
First-phase ejection fraction (${\mathrm{EF}}_{1}$) [83]	${\mathrm{EF}}_{1}$(%) = [(EDV – $V_{1}$)/EDV] × 100	- Can predict cardiac worsening in patients with ≥moderate AS better than other parameters including the global longitudinal strain, the AV area index, mean pressure gradient, stroke volume index (SVi) and trans-aortic flow rate.
	$V_{1}$ = the LV volume at the time point of the peak blood flow across the AV	- Appeared helpful for optimal timing of AV replacement in asymptomatic patients with severe AV narrowing.
Total systemic arterial compliance (SAC) [79, 80, 81, 82]	SAC = SVi/PP	- In older patients with AS, reduced SAC (≤0.6 mL/$m^{2}$/mmHg) has an additive effect on LV pressure overloading during ejection associated with a major impact on the deterioration of LV function.
	SVi = stroke volume index	- SAC appears useful for differentiation between the relative contribution of AS and arterial stiffness on LV pressure overloading.
	PP = brachial pulse pressure*	
“Valvulo-arterial impedance” ($Z_{\text{va}}$) [80, 81]	$Z_{\text{va}}$ = (SAP + ${\mathrm{MG}}_{\text{net}}$)/SVi	- $Z_{\text{va}}$ represents the valvular and arterial factors that oppose LV ejection by absorbing the mechanical energy developed by the LV. Its calculation allows the estimation of the global LV afterload in AS patients.
	SAP = systolic arterial pressure	- $Z_{\text{va}}$ ≥5.0 mmHg/mL/$m^{2}$ indicates increased afterload that exceeds the limit of LV compensatory mechanisms and, therefore, leads to afterload mismatch with subsequent LV systolic dysfunction.
	${\mathrm{MG}}_{\text{net}}$ = mean net gradient across the AV	
Energy loss index (ELI) [80]	ELI = [(EOA × $A_{\text{A}}$)/($A_{\text{A}}$ – EOA)]/BSA	- Useful for a better classification of patients with AS, based on both the severity of AV narrowing (severe AS defined as ELI ≤0.55 ${\mathrm{cm}}^{2}$/$m^{2}$) and the degree of SAC alteration.
	EOA = the effective orifice area of the AV	- ELI, together with SAC and $Z_{\text{va}}$, can identify patients who could benefit from AV replacement despite reduced SAC values.
	$A_{A}$= aortic cross-sectional area	
Global work index (GWI) [86, 87, 88, 89, 90, 91]	Calculated by software from the global longitudinal strain (GLS), blood pressure values, and PW Doppler-derived time measurements of valvular events.	- GWI quantifies the indexed total work performed by the LV throughout the entire mechanical systole including both IVC and IVR (corresponds to the myocardial energy translated into mechanical energy between MV closure and opening).
		- This new parameter has the advantage of incorporating information on afterload, through the interpretation of strain in relation to dynamic non-invasive LV pressure.
Global constructive work (GCW) [87, 88, 89, 90, 91]	It is calculated by the same software, using some of the measurements which were also used for calculation of GWI	- GCW is the LV work that contributes to LV ejection and is performed by shortening (positive work) during IVC and systole or by lengthening (negative work) in IVR.
		- It quantifies the energy consumed by the myocardium that effectively contributes to cardiac output by facilitating LV ejection.
Global wasted work (GWW) [87, 88, 89, 90, 91]	It is calculated also by the same software, using some of the measurements which were used for calculation of GWI	- Represents the LV work that does not contribute to ejection.
		- It quantifies the energy consumed by the myocardium that is wasted and does not contribute to the cardiac output (CO).
Global work efficiency (GWE) [87, 88, 89, 90, 91]	GWE (%) = [GCW/(GCW + GWW)] × 100	- GWE is the ratio between constructive work and total (constructive and wasted) work, which reflects the net percentage of the performed myocardial work, which is actually translated into CO.

MR, mitral regurgitation; LV, left ventricle; LVEF, left ventricular ejection fraction; ESV, end-systolic volume; PW, pulsed-wave; AV, aortic valve; AS, aortic stenosis; IVC, isovolumic contraction; IVR, isovolumic relaxation; BSA, body surface area; MV, mitral valve. * PP, difference between systolic and diastolic arterial blood pressure.

### 6.2 Right Ventricular Evaluation in Relation to Loading Conditions

The presence of a distinctively high sensitivity of ventricular size, geometry and function to pressure overloading associated with a relatively high tolerance to volume overloading are major particularities of the RV with a relevant impact on the reliability of its evaluation [9, 50, 96, 97, 98, 99]. Therefore, the evaluation of the RV and LV differ not only in terms of the preferred techniques and measured parameters but even more importantly in the interpretation of the measurements in context with their particularities and the instantaneous hemodynamic loading conditions [100, 101].

#### 6.2.1 Role in Estimation of the RV Recovery Potential and Early Recognition of Impending RV Failure

Assessment of the RV in relation to its actual loading conditions and prediction of both RV reverse remodeling and functional improvement in case of a reduction of its hemodynamic overloading is particularly helpful in decision-making before heart transplantation (HTx) and VAD implantation, as well as for optimal timing of lung transplant listing for patients with refractory end-stage precapillary PH [66, 102].

End-stage HF involves both ventricles, even if its initial cause was left-sided heart disease. Although LV assist devices (LVADs) provide better survival and quality of life than biventricular assist devices, it must also be considered that RVF associated with increased morbidity (more often renal, hepatic or multi-organ failure) and mortality can occur in about 25% of LVAD recipients, even if LVAD implantation is later followed by a complementary RV assist device (RVAD) implantation [103, 104]. Therefore, patients who require a long-term biventricular assist device, as well as those who require a temporary RVAD in addition to the LVAD should be identified already before surgery or at the latest intraoperatively [102, 105]. In the meantime, it has become obvious that an Echo can be a cornerstone for major decision-making processes prior to, as well as during, VAD implantation surgery [105, 106]. Those patients who had an unfavorable clinical course of RV function after LVAD implantation, revealed already pre-operatively significant differences in Echo-derived parameter values related to right atrial (RA) and RV size, geometry and function [106, 107, 108, 109, 110]. Nevertheless, such anatomical and functional RV alterations in LVAD candidates could not be recognized in all studies as significant risk factors for RVF during LVAD support [100, 101, 102]. The vast majority of Echo-derived variables identified as risk factors for RVF were found alone not able to predict neither RVF nor provide freedom from RVF after LVAD implantation [101, 106].

RVF induced by myocardial pressure overloading is the main cause of death in patients with severe PAH (i.e., mortality rate up to 40% in PAH patients with acute RHF) and chronic thromboembolic PH [26, 109]. With the continuous prolongation of waiting-times for lung transplantation (LTx), timely prediction of no longer reversible RVF in LTx candidates is crucial for the optimal timing of listing procedures, and therefore, the identification of significant prognostic predictors is a major goal [110, 111, 112].

#### 6.2.2 Attempts for RV Evaluation in Relation to Loading Conditions

Two-dimensional echocardiography (2D-Echo) is the main working tool for routine clinical evaluation of the RV anatomy and function. However, the utility of RV volume measurements necessary for the calculation of the EF appeared unreliable [100, 101]. Accordingly, RVEF calculations derived from 2D-Echo measurements are no longer recommended, neither for scientific research nor for clinical use [25, 56]. Because 3D-Echo provides better reproducibility and higher accuracy of RV volume measurements, it can replace 2D-Echo for these measurements [56, 64]. However, 2D-Echo remains particularly useful for the assessment of RV and RA size and function, detection and quantification of tricuspid regurgitation (TR), as well as for the measurement of the pressure gradient between the RV and the RA (Δ$P_{\text{RV-RA}}$) [100, 101, 102, 106, 108]. Parameters like RV fractional area change (${\mathrm{FAC}}_{\text{RV}}$) and TAPSE, and especially the 2D-STE-derived RV and RA strain and strain rate parameters can provide important functional details.

Although a large number of non-invasively and invasively derived individual parameters were tested over the time for their diagnostic and prognostic value in different pathological conditions with relevant involvement of the right-sided heart, there is still controversy about the reliability of the individual parameters used in the clinical praxis. The main reason for this controversy lies in the fact that all right-sided anatomical and functional parameters are load-dependent [21, 100]. Given the particularly high load dependency of RV size, geometry and pump function, it is necessary to consider this fundamental aspect in the interpretation of all collected data related to the evaluation of the right-sided heart. Because no single parameter, regardless the method (e.g., Echo, MRI or CT) used for its measurement, can alone reveal the overall picture of RV dysfunction, it is necessary to carry out multiparametric assessments and to also apply integrative approaches using combinations of parameters which should always also include details about the RV hemodynamic loading conditions [21, 68, 113]. In the past few years, the potential usefulness of various parameters and indices of ventricular myocardium adaptability to load referring to different concepts like ratios of functional parameters and load, RV-PA coupling, or indices reflecting the ability of the RV to overcome increased PVR was also investigated [27, 49, 68]. Over the past few years, several indices of load adaptability referring to different concepts like simple ratio of function and load, RV-PA coupling, or indices assessing the ability of the RV to overcome increased PVR have been proposed [27, 49, 68]. There was a general tendency to create parameter combinations and indices from measurements obtained either by invasive or non-invasive examinations.

The best known index obtained from invasive measurements is the right heart catheterization derived stroke work index, which is calculated by the formula: right heart catheterization (RHC) derived RV stroke work index (${\mathrm{RVSWi}}_{\text{RHC}}$)= (mPAP–mRAP) × SVi, where mPAP is the mean PA pressure, mRAP the mean RA pressure and SVi the mean SV index [113, 114, 115, 116]. This parameter was found useful for the prediction of a short-term severe worsening of RV function in pre-capillary PH, as well as for pre-implant prediction of post-implant RVH in LVAD candidates with secondary RV dilation and dysfunction [113, 114, 115, 116]. In LVAD candidates, although this index was identified as an independent predictor for RVF after LVAD implantation with a higher predictive value in comparison with conventional Echo parameters like ${\mathrm{FAC}}_{\text{RV}}$ or RV outflow tract fractional shortening (which were also identified as independent predictors), its predictive value was lower than that revealed by the STE-derived RV free wall longitudinal strain (RVFWLS), which is undoubtedly an afterload dependent parameter [113]. Thus, this observation can also be considered as an indication of the existence of limitations for the use of solely RHC-derived measurements which provide no direct information, neither about the RV geometry and size, nor about its contractile abilities. In principle, it is also possible to calculate the RVSWi by using conventional Echo-derived measurements because the mean pressure gradient mPAP– mRAP can be replaced by the continuous-wave Doppler-derived mean RV–RA pressure gradient obtained from the velocity-time integral (VTI) of TR, whereas the Echo-derived measurements necessary for the SV calculation are easily obtainable [113, 116, 117].

Most of the tested integrative approaches based only on Echo-derived parameters appeared more or less limited by the lack of more precise hemodynamic data. Nevertheless, these approaches have the advantage of being particularly suited for close monitoring of outpatients. Undoubtedly, the most optimal integrative approaches would necessitate both non-invasively obtained RV measurements (derived from different Echo techniques, CT or MRI) and invasively obtained hemodynamic details during RHC, because most of the tested approaches based only on Echo-derived parameters appeared limited due to the lack of more precise hemodynamic data [15, 16, 21]. Thus, with the exception of the above mentioned RVFWLS, the RHC-derived RVSWi was found more useful than the currently used Echo-derived parameters for the evaluation of the RV in end-stage congestive HF [113, 114]. The correlation between the RV stroke work index (${\mathrm{SWI}}_{\text{RV}}$) calculated from Echo-derived and RHC-derived measurements can also be poor [114], which is quite understandable given the lack of precise hemodynamic data provided by the Echo-derived parameters as well as the lack of information about the RV size, geometry and myocardial contractile properties provided by RHC-derived parameters.

During the last few years, several complex Echo-derived indexes suggested as possible surrogates for the RHC-derived RVSWi, as well as different Echo-derived composite variables which incorporate either RV myocardial displacement and load, or velocity of myocardial shortening and load, were tested for their usefulness for both the evaluation of RV remodeling and dysfunction, and the prediction of impending severe RV failure [27, 49, 113, 114, 115, 116, 117, 118].

A simplified composite Echo-derived index was proposed as a surrogate for the ${\mathrm{SWI}}_{\text{RV}}$ is the “RV contraction-pressure index” (RVCPI), which is derived as RVCPI = TAPSE × Δ$P_{\text{RV-RA}}$ [117, 119, 120]. The RVCPI showed a close correlation with the RHC-derived ${\mathrm{SWI}}_{\text{RV}}$ and a high sensitivity and specificity to predict depressed ${\mathrm{SWI}}_{\text{RV}}$ [117]. In a prospective study including patients with advanced congestive HF, a multivariate analysis identified the presence of a low RVCPI as the best predictor of outcome, whereas neither TAPSE or ${\mathrm{FAC}}_{\text{RV}}$, nor TAPSE/systolic PAP or ${\mathrm{FAC}}_{\text{RV}}$/systolic pulmonary arterial pressure (PAP) revealed significant predictive values [119]. The usefulness of the RVCPI as an independent predictor of short-term postoperative patient outcomes (including RV failure) after LVAD implantation was also confirmed in one study [120]. Another Echo-derived surrogate for the invasively obtained ${\mathrm{SWI}}_{\text{RV}}$ (i.e., calculated from RHC-derived measurements), designed by the authors as “RV stroke work” (${\mathrm{RVSW}}_{\text{Echo}}$), also showed a close correlation with the RHC-derived ${\mathrm{SWI}}_{\text{RV}}$ [118]. This Echo-derived RVSW incorporates the SV and load and is calculated as: ${\mathrm{RVSW}}_{\text{Echo}}$ = 4 × [TR jet peak velocity]^2^ × [pulmonary valve-area × VTI], where VTI is the velocity-time integral of the systolic transpulmonary jet [118].

During the last decade, certain Echo-derived composite indices which include either longitudinal movement of a RV myocardial component and load (i.e., TAPSE/systolic PAP and TAPSE/PVR) or velocity of myocardial shortening (i.e., velocity of deformation) and load (i.e., afterload-corrected peak systolic longitudinal strain rate) appeared also useful for assessment of RV contractile function [5, 8, 121, 122, 123, 124, 125, 126, 127].

The TAPSE/systolic PAP ratio is a simplified approach to assess RV contraction by plotting longitudinal myocardial shortening vs. the force generated for overcoming the imposed load [121, 122, 123, 124]. This parameter can facilitate therapeutic decision-making processes and prognostic assessments in patients with RV dysfunction, and, based on its high correlation with invasively evaluated RV systolic elastance/arterial elastance TAPSE/systolic PAP was also proposed as an index of RV-PA coupling [124]. Several studies demonstrated a high reproducibility of the necessary measurements and this index appeared able to predict mortality in patients with HF due to primary impaired LV function and also in patients with severe PAH [68, 125]. However, as mentioned above the significant predictive value of mortality in patients with HF originating from primary impaired LV function was not confirmed by all studies [119].

The RV ejection efficiency (RVEe), defined as RVEe = TAPSE/PVR, is another composite variable which was proposed as a non-invasive index of RV-PA coupling [12]. Thus, using TAPSE as a surrogate for RV ejection and the Echo-derived PVR as a surrogate for the RHC-derived PVR, the RVEe is easily calculable with the formula: PVR = TR peak velocity/RV outflow tract velocity-time integral. The calculation of the RVEe might be appropriate for the assessment of RV systolic function. However, further studies will be necessary to determine whether the Echo-derived RVEe can be indeed useful for the assessment of RV function. A limitation of this index is its decreasing reliability with the aggravation of the TR in patients with high afterload-induced severe RV dilation. It is well-known that advanced TR is a confounding factor that can affect the use of TAPSE for assessing RV function [100, 102, 126, 127].

The ratio of SV/RV end-systolic volume (SV/RVESV) was also recommended as a surrogate for the RV-PA coupling, defined as the ratio between ventricular maximal elastance and arterial elastance (i.e. Emax/Ea), which could therefore be useful in the evaluation of myocardial contractility corrected for afterload. The major limitations of SV/RVESV are the fundamentally wrong suppositions that the relationship between the RV end-systolic pressure and RVESV is linear and crosses the origin, and that, also for the RV, the end-systolic elastance (Ees) coincides with Emax [128]. Another weakness of SV/RVESV is the inaccurate measurement of RV volumes with 2D-Echo. Given that the 2D-Echo-derived RV area measurements are much more reliable than the RV volume measurements, and one study [68] has already confirmed the potential usefulness of the RV area change/RV end-systolic area ratio as a prognostic marker in patients with severe PAH, this simple approach could be more useful in a clinical setting.

The “afterload-corrected peak systolic global longitudinal strain rate” (GLSR), based on the relationship between RV myocardial shortening-velocity and RV load, which is calculated by multiplying the measured systolic GLSR value with the Δ$P_{\text{RV-RA}}$, is an easy, obtainable and reproducible combined parameter for the evaluation of RV contractile function in relation to loading conditions [27, 49]. Due to the load-dependency of myocardial shortening velocity, the GLSR will decrease simultaneously with the increase of the RV systolic pressure. Thus, as long as the RV contractile function remains stable, also the combined parameter GLSR × Δ$P_{\text{RV-RA}}$ remains relatively stable. However, once the afterload increase overwhelms the ability of the RV to adapt its pump function correspondingly (i.e., afterload mismatch), any further reduction of the GLSR will be associated with an increase of the RA pressure with a corresponding reduction of the Δ$P_{\text{RV-RA}}$, even before the RV systolic pressure will finally also decrease as a result of the ongoing maladaptive ventricular remodeling and deterioration of RV myocardial contractility [27, 49]. The load-corrected peak GLSR can be therefore more helpful for the assessment of RV contractile function than the peak GLSR alone and has the advantage to include also the impact of TR on the RV function.

**Fig. 2.**
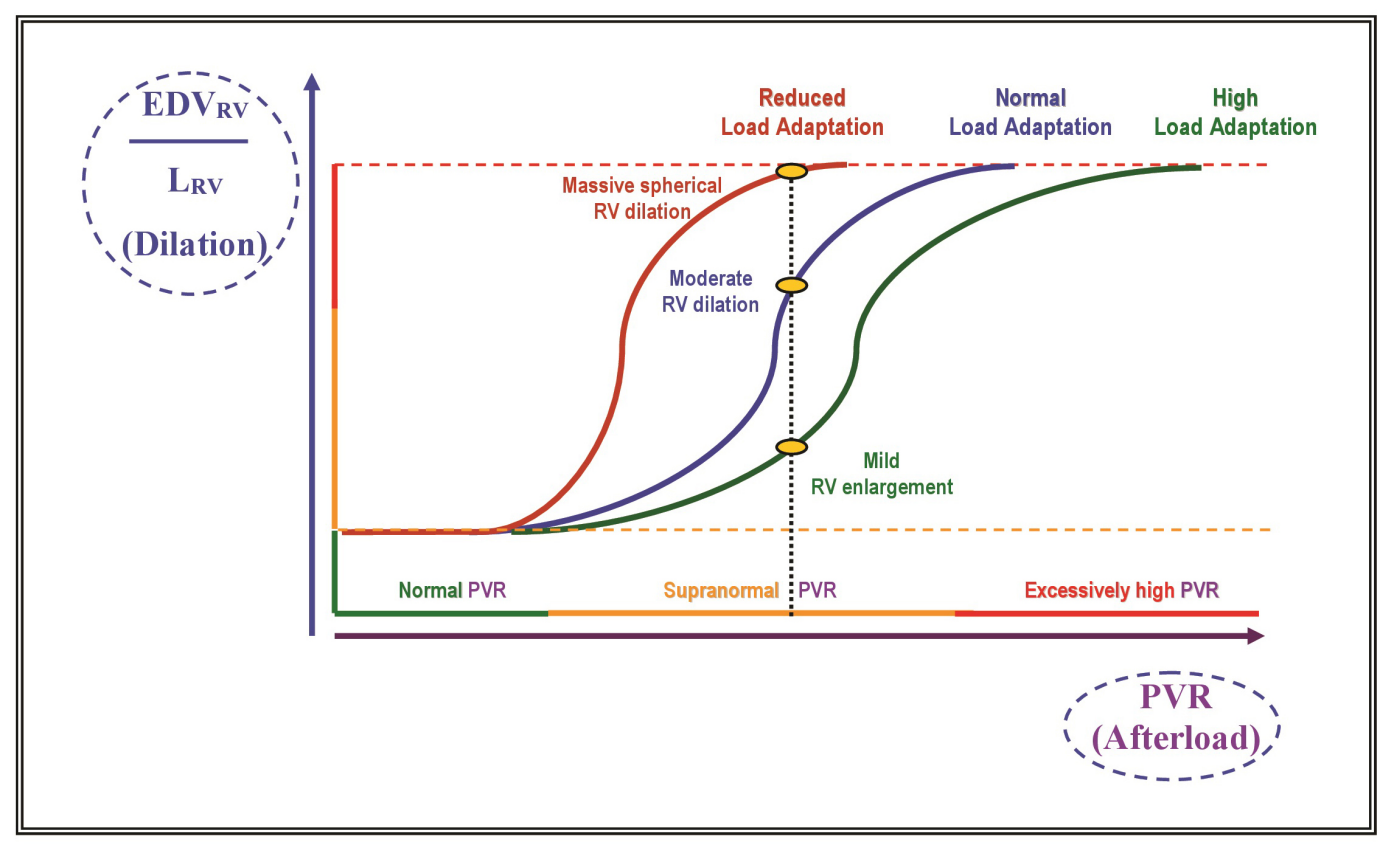
**Right ventricular adaptation to increasing afterload**. PVR, pulmonary vascular resistance; RV, right ventricle; ${\mathrm{EDV}}_{\text{RV}}$, RV end-diastolic volume; $L_{\text{ED}}$, end-diastolic long-axis lengths; $L_{\text{RV}}$, RV long-axis length. RV adaptation to increasing afterload is describable by the ratio between the right heart catheterization (RHC) derived PVR and the RV dilation (${\mathrm{EDV}}_{\text{RV}}$/$L_{\text{RV}}$) assessed by echocardiography. PVR × $L_{\text{RV}}$/${\mathrm{EDV}}_{\text{RV}}$ can be therefore considered as an RHC- and echocardiography-derived load adaptation index. As shown in the figure, at identical supranormal PVR values, reduced adaptation is reflected by massive spherical dilation (i.e., excessively high ${\mathrm{EDV}}_{\text{RV}}$/$L_{\text{RV}}$) and therefore by a correspondingly reduced PVR × $L_{\text{RV}}$/${\mathrm{EDV}}_{\text{RV}}$. Using the Doppler echocardiography-derived tricuspid regurgitation velocity-time integral (${\mathrm{VTI}}_{\text{TR}}$) as a surrogate of hemodynamic load instead of the PVR and the RV end-diastolic area ($A_{\text{ED}}$) instead of the not reliably measurable ${\mathrm{EDV}}_{\text{RV}}$ allows the non-invasively obtainment of a simple highly reproducible dimensionless RV load adaptation index: ${\mathrm{LAI}}_{\text{RV}}$ = [${\mathrm{VTI}}_{\text{TR}}$ (cm) × $L_{\text{ED}}$ (cm)]/$A_{\text{ED}}$ (${\mathrm{cm}}^{2}$).

**Fig. 3.**
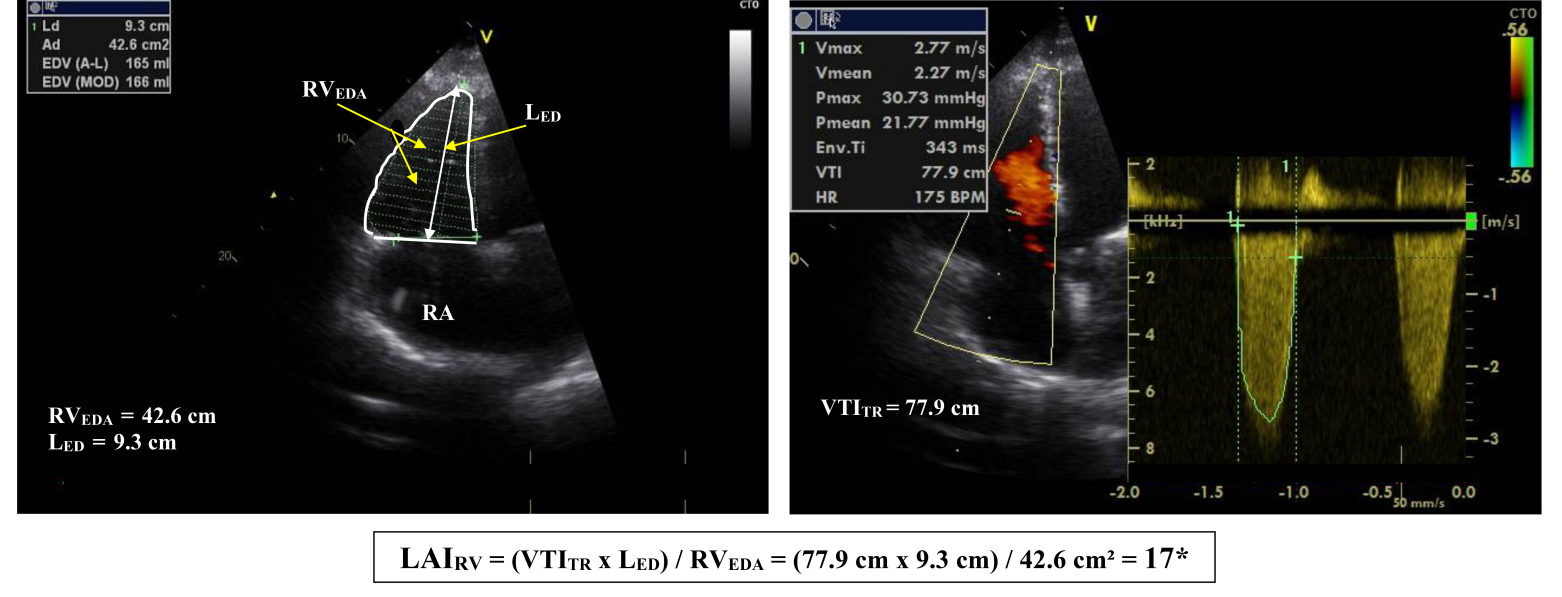
**Calculation of the right ventricular “load adaptation index” from 2D-Echo-derived measurements in a patient with advanced heart failure due to primary impaired left ventricular function**. ${\mathrm{RV}}_{\text{EDA}}$, right ventricular end-diastolic area (in the apical 4-chamber view); $L_{\text{ED}}$, end-diastolic long axis length; ${\mathrm{VTI}}_{\text{TR}}$, velocity-time integral calculated from the continuous wave Doppler-derived measurements of the tricuspid regurgitation velocity; RA, right atrium; HR, heart rate; ${\mathrm{LAI}}_{\text{RV}}$, lead adaptation index of the right ventricle. * Values ≤18 reflect a highly reduced RV adaptability to persistent hemodynamic overloading, whereas values ≤15 indicate a severely impaired adaptability of the RV to afterload which could be insufficient to provide a sufficient ejection of blood even after normalization of the afterload (e.g., after LVAD implantation in advanced LV failure or lung transplantation in refractory pulmonary arterial hypertension).

A different approach for the assessment of RV adaption to pathologically elevated afterload is provided by the Echo-derived “RV load-adaptation index” (${\mathrm{LAI}}_{\text{RV}}$), a composite variable which reflects the relationship between RV hemodynamic load and RV dilation [27, 49, 68]. The theoretical basis for the combination of the individual components of that index is the fact that in patients with similar resistance to the blood flow in the pulmonary circulation, less RV dilation indicates better adaptation to high afterload (Fig. 2). As shown in Fig. 3, using for the ${\mathrm{LAI}}_{\text{RV}}$ calculation the easily measurable TR velocity-time integral (${\mathrm{VTI}}_{\text{TR}}$) as a surrogate of the hemodynamic load and the RV end-diastolic area ($A_{\text{ED}}$) instead of the not reliably measurable RVEDV for obtaining together with the end-diastolic long axis lengths ($L_{\text{ED}}$) a reliable RV size-geometry index, allows the obtainment of a highly reproducible dimensionless index: ${\mathrm{LAI}}_{\text{RV}}$ = [${\mathrm{VTI}}_{\text{TR}}$ (cm) × $L_{\text{ED}}$ (cm)]/$A_{\text{ED}}$ (${\mathrm{cm}}^{2}$) [27, 49, 102]. The use of the ${\mathrm{VTI}}_{\text{TR}}$ as a surrogate of the RV hemodynamic load and not the Δ$P_{\text{RV-RA}}$, which is calculated from the mean velocity of the TR jet allows not only the obtainment of a dimensionless index, but it has also the advantage of including the duration of the afterloading during the RV systole [27, 49, 102]. Inclusion of the end-diastolic and not the end-systolic RV area and long-axis measurements into the ${\mathrm{LAI}}_{\text{RV}}$ calculation formula is more appropriate, especially in advanced RV overloading, because RV dilation is more reliably quantifiable in the end-diastole (particularly in the presence of relevant TR, which leads to underestimation of the RV dilation in the end-systolic phase) [27, 102]. A small RV area relative to the long-axis length (i.e., unaltered RV size and geometry) in a person with a high ${\mathrm{VTI}}_{\text{TR}}$ (i.e., high RV systolic pressure without elevated RA pressure) gives a higher ${\mathrm{LAI}}_{\text{RV}}$ value, which strongly suggests an unrestricted adaptation to load (i.e., capability to rise the RV systolic pressure without either a significant RV dilation, or a relevant elevation of the RA pressure) indicating a good or at least adequate RV contractile function, and also the ability of the RV to improve its pump function after reduction of the pressure overloading [27, 102]. A spherical RV dilation indicated by a large RV area relative to its long-axis length despite a rather low ${\mathrm{VTI}}_{\text{TR}}$ value, which points to a greater increase in RA pressure than in RV systolic pressure, will generate a low ${\mathrm{LAI}}_{\text{RV}}$ which reveals the presence of a poor adaptation to hemodynamic overloading (disproportionally severe RV dilation despite a relatively low RV pressure load indicating also a reduced RV systolic function) indicating also a reduced RV myocardial contractility. It was found that ${\mathrm{LAI}}_{\text{RV}}$ values <15 suggest an excessively low RV adaptability to load which can be incapable to prevent RVF even in the presence of a normal PVR [49, 68, 102, 129].

Recently, the Echo-derived RV global work efficiency (RV-GWE) was found useful for predicting the risk of early RHF after LVAD implantation [130]. In this small study, the predictive value of the RV global work efficiency was superior to that of both the TAPSE/systolic PAP and the RVFW longitudinal strain/sPAP ratio.

More recently, 3D-STE data were used to assess the relationship between RV remodeling and afterload (i.e., RV end-systolic volume index and systolic PAP) [131]. Regression analysis between the systolic PAP and RV end-systolic volume index appeared to be able to distinguish adapted, adapted-remodeled and adverse-remodeled RV from one another [131].

Table 2 (Ref. [5, 8, 12, 27, 49, 68, 117, 118, 119, 120, 121, 122, 123, 124, 128, 130]) provided an overview of major Echo-derived combined parameters and indices for evaluation of RV myocardial responses to pressure and/or volume overloading.

**Table 2.** **Overview on major echocardiography-derived combined parameters and indices for evaluation of right ventricular myocardial responses to pressure and/or volume overloading**.

Parameters	Calculation	Particularities and clinical usefulness
RV contraction-pressure index (RVCPI) [117, 119, 120]	RVCPI = TAPSE × Δ$P_{\text{RV–RA}}$	- The RVCPI revealed a close correlation with the RHC-derived ${\mathrm{SWI}}_{\text{RV}}$ plus high predictability of depressed ${\mathrm{SWI}}_{\text{RV}}$.
	TAPSE = tricuspid annular peak systolic excursion	- Was found to be an independent predictor of early RVF after LVAD implantation.
	Δ$P_{\text{RV–RA}}$ = pressure gradient between the RV and RA	- In a multivariate analysis, a low RVCPI was identified as the best predictor of outcome, whereas neither TAPSE/sPAP nor ${\mathrm{FAC}}_{\text{RV}}$/sPAP revealed significant predictive values.
RV stroke work (RVSW) [118]	RVSW = 4 × [TRj peak velocity]^2^ × [PVA × VTI]	- By incorporating the SV and load, it revealed a strong correlation with the RHC-derived ${\mathrm{SWi}}_{\text{RV}}$.
	TRj = tricuspid regurgitation jet	- Direct calculation of the RV stroke volume can be challenging due to the difficulties to counteract the angle dependency of systolic flow VTI measurements along the PVA.
	PVA = pulmonary valve area	
TAPSE/sPAP [121, 122, 123, 124]	Calculated from conventional echo-derived measurements.	- Easily obtainable useful parameter for estimation of RV performance by assessing the relationship between longitudinal displacement and load, which correlates well with the invasively evaluated RV systolic elastance/arterial elastance.
		- Was found useful for prediction of cardiovascular mortality in patients with HF induced by LV dysfunction, as well as in patients with advanced PAH, but this usefulness could not be confirmed in all studies.
RV ejection efficiency (RVEe) [12]	RVEe = TAPSE/PVR	- A limitation is its decreasing reliability with the aggravation of the TR.
	Echo-derived PVR = TR peak velocity/${\mathrm{VTI}}_{\text{RVOT}}$	- Is clinical usefulness is currently not established.
SV/RVESV ratio [128]	Stroke volume/RV end-systolic volume	- Is considered as a surrogate for the RV-PA coupling which is defined as Emax/Ea (i.e., ratio of end-systolic ventricular elastance and arterial elastance). It facilitates the estimation of myocardial contractility corrected for afterload.
		- Its limitations are the incorrect inherent assumptions that the relationship between the RV end-systolic pressure and RV endsystolic volume is linear and crosses the origin and that Ees coincides with Emax, as well as its as the not very reliable calculation of RV volumes from 2-dimensional echocardiography-derived parameters.
RVArea change/ESA [68]	RV area change/RV end-systolic systolic area	- It was proposed as a surrogate for RV-PA coupling. One study has already confirmed its potential usefulness for the prognostic assessment of patients with advanced PAH.
Afterload-corrected peak GLSR [5, 8, 27, 49]	Afterload corrected peak GLSR = GLSR × Δ$P_{\text{RV-RA}}$, where GLSR is the global longitudinal strain rate, and Δ$P_{\text{RV-RA}}$ the pressure gradient between the RV and RA.	- Easily obtainable and reproducible combined variable reflecting the relation between RV myocardial velocity of shortening and RV loading conditions. This combined parameter was found more useful for the assessment of RV contractile function than the peak GLSR as an individual parameter.
		- Has the advantage of also including the impact of TR on RV function.
		- Revealed a high ability to predict in LVAD candidates the development or aggravation of RHF after LVAD implantation, as well as a high predictive value for imminent worsening of RV function in potential lung transplant candidates with precapillary PH.
RV load-adaptation index (${\mathrm{LAI}}_{\text{RV}}$ ) [5, 8, 27, 49, 68]	${\mathrm{LAI}}_{\text{RV}}$ = [${\mathrm{VTI}}_{\text{TR}}$ (cm) × $L_{\text{ED}}$ (cm)]/$A_{\text{ED}}$ (${\mathrm{cm}}^{2}$)	- Simple and highly reproducible parameter which reflects the relationship between RV hemodynamic load and RV dilation which appeared preoperatively highly predictive for RV function after implantation of a LVAD.
		- Appeared highly predictive value for imminent RV failure in lung transplant candidates with pre-capillary PH.
RV global work efficiency (GWE) [130]	GWE (%) = [GCW/(GCW + GWW)] × 100	- Was found useful for predicting the risk of early RHF after LVAD implantation.
		- Its predictive value was found superior to that of both the TAPSE/sPAP and the RVFW longitudinal strain/sPAP ratio.

RV, right ventricle; RHC, right heart catheterization; SWI, stroke work index; sPAP, systolic pulmonary arterial pressure; FAC, fractional area change; VTI, velocity-time integral; SV, stroke volume; HF, heart failure; PAH, pulmonary arterial hypertension; TR, tricuspid regurgitation; PA, pulmonary artery; Ees, end-systolic elastance; PH, pulmonary hypertension; RHF, right heart failure; RVFW, RV free wall; GCW, global constructive work; GWW, global wasted work; RA, right atrium; RVF, RV failure; ESA, end-systolic area; LVAD, left ventricular assist device; ${\mathrm{SWi}}_{\text{RV}}$, RV stroke work index; PVR, pulmonary vascular resistance; RVESV, RV end-systolic volume; RVOT, right ventricular outflow tract; GLSR, global longitudinal strain rate; $L_{\text{ED}}$, end-diastolic long-axis lengths; $A_{\text{ED}}$, end-diastolic area.

## 7. Conclusions

Assessment of ventricular dysfunction and prediction of its further course, selection of the most appropriate therapeutic approaches, as well as monitoring of therapy results, are crucial to the successful management of patients with HF. However, despite the important progress achieved in medical technology with a corresponding improvement of diagnostic methodologies (particularly for cardiac imaging) there are still substantial challenges, particularly those related to the load dependency of ventricular morphology and myocardial contractile function. The latter fact explains why no single parameter is able to alone reveal the real picture of ventricular dysfunction.

Given that the use of a single parameter is inadequate, it is necessary to perform multiparametric evaluations and to also apply integrative approaches using parameter combinations which include details about the ventricular loading conditions. This is particularly important for the evaluation of RV dysfunction because of its remarkably high sensitivity to supernormal afterload. In this regard, the existence of certain reluctances towards the implementation of such parameter combinations in the routine clinical praxis is difficult to understand.

Among the non-invasive attempts to evaluate ventricular function in connection with its current loading conditions, the relationship between ventricular contraction (e.g., myocardial displacement or deformation) and pressure overload, the relationship between ejection volume or ejection velocity and pressure overload, as well as the relationship between ventricular dilation and pressure overload were found useful for therapeutic decision making [21, 27, 68, 69]. However, based on the available evidence, currently it is not possible to establish a reliable hierarchy of combined parameters based on their reliability for evaluation of the LV and RV in relation to their loading conditions. Given the crucial impact of interconnected myocardial remodeling and contractility on the ability of a ventricle to overcome hemodynamic overload, it would be more beneficial to use combined parameters reflecting both the relationship between pump function and afterload, and the relationship between ventricular overloading and remodeling responses for clinical decision-making (particularly in the case of severe RV dysfunction). The latter approach would also have the advantage of diminishing the negative impact of TR on the reliability of parameters which reflect the relationship between pump function and afterload by the concurrent use of the RV load adaptation index whose reliability even increases with the progression of TR [27, 100, 102].

In the future it will be necessary to pay more attention to the load dependency of ventricular pump function and to take into consideration its impact on the evaluation of the severity and the prognostic relevance of myocardial dysfunction. Further sustained efforts to provide more evidence for its practical importance by intensifying the clinical research in this field could be a solid basis for achieving this goal.
